# Dental Public Health Landscape: Challenges, Technological Innovation and Opportunities in the 21st Century and COVID-19 Pandemic

**DOI:** 10.3390/ijerph17103636

**Published:** 2020-05-21

**Authors:** Marco Mascitti, Giuseppina Campisi

**Affiliations:** 1 Department of Clinical Specialistic and Dental Sciences, Marche Polytechnic University, 60126 Ancona, Italy; 2 Department of Surgical, Oncological and Oral Sciences, University of Palermo, 90127 Palermo, Italy

In response to the 2008 economic and financial crisis and to its effects on healthcare systems, dental care has become unaffordable for many people, and a huge number of patients worldwide are avoiding or skipping necessary dental treatments. Furthermore, the progressive aging of the global population and the associated increase in general and dental care needs contribute to concerns regarding the sustainability of healthcare systems. These trends highlight the urgent need for a new sustainable and efficient model of dental care [1]. In this regard, the adoption of modern technology in dentistry, also called by many as “digital dentistry”, is the most promising strategy for reshaping the landscape of oral healthcare. The tumultuous technological progress that we have been experiencing in recent years, in particular since the onset of the Web 2.0 in 2004, has now pervaded every aspect of daily living. Several promising digital technologies, such as the augmented reality, virtual reality, and internet of things are only the most recent and striking examples of this revolution, which goes far beyond purely technological upheavals.

Moreover, the World Health Organization repeatedly advocate for change, promoting the development and diffusion of public health practices supported by both information and communications technologies (e-Health) and mobile devices (m-Health) [2]. By focusing on the definition of Dental Public Health (DPH) as “the science and art of preventing and controlling dental diseases and promoting dental health through organized community efforts” [3], we realize how technological innovations could help achieve these goals by providing useful tools.

Let it be clear that when we talk about “digital dentistry”, we are not only referring to micro-trends in digital radiography, intra-oral scanners, and digital dental workflow. Above all, the implementation of modern technology in dentistry is an opportunity to make a real paradigm shift from a “disease-centered” model to a “patient-centered” care approach. Indeed, one aspect that has attracted considerable attention is the possibility for a change of approach toward well-known issues in DPH [2].

The first and foremost goal is to realize a revolution in oral health prevention. To date, the efforts of preventive dentistry help to reduce the worldwide prevalence of the most common dental diseases. However, it has always been conceived within the context of a traditional reactive healthcare system that treats dental disease only after their appearance. Modern technologies offer the opportunity to change the way we treat oral diseases, encouraging patients to be more active drivers of their own health. For example, mobile technology could be used to monitor actual oral hygiene behaviors, creating effective feedback systems to support oral health [1].

Another expanding field with enormous potential for the future is called teledentistry, defined as the remote provision of oral health care through the use of information technology [4]. This approach offers a wide variety of clinical applications, ranging from special needs dentistry to oral medicine [5]. Furthermore, teledentistry applications can be particularly useful in those countries where geographical distance hinders the access to oral healthcare systems for millions of people. Not to mention the remarkable advances in oral cancer diagnosis through the use of new devices [6] or the promising future of salivaomics, namely the use of saliva as diagnostic fluid for oral and systemic diseases [7].

People with disabilities most likely need the improvement of a DPH system among others. The efforts to secure an equal access to oral health care services are markedly heterogenous throughout the world. Despite the existence of long-term social policies directed towards reducing health inequalities, disabled people have poorer oral health, even in developed countries. Considering patients with special needs, cancer patients are the ones who will require more than anyone else a change in traditional dental practices. Indeed, thanks to an increase in their survival, there is a growing demand for oral care in order to improve the quality of life. Although general health problems and disabilities in these patients are prominent, oral health maintenance should not be overlooked. The management of these complex patients, that are at higher risk for oral complications, require a multidisciplinary approach, which could be facilitated by the adoption of modern technologies. In particular, the development of hub-and-spoke teledentistry programs can improve healthcare quality and patient satisfaction [8].

The role of technological advances in DPH will certainly have consequences in the way of facing the new challenges that are arising and that we cannot postpone addressing it. The most arduous challenge for DPH in the next decades is certainly the ageing population. According to WHO, by 2050, the number of over 60s will exceed a fifth of the global population, with inevitable economic and social consequences [9]. As for dental specialists, a promising approach could be the extensive use of teledentistry in residential and nursing homes combined with a home dental service for frail patients, all operating in a hub-and-spoke system.

The current coronavirus pandemic is the perfect example of an unexpected emergency that can put the worldwide health systems in crisis, even in the most developed countries. Considering that COVID-19 is a highly communicable disease, teledentistry is ideal for the remote patient management, contributing to the slowdown of the virus transmission.

However, despite all promising prospects, we must highlight the presence of a number of economic, ethical, and social concerns that have not yet been sufficiently investigated. The first of these concerns is the real impact and effectiveness of new technologies on population health. Indeed, a “technologically enhanced” DPH should target more precisely those groups of people who need it most, otherwise it risks producing limited effects, despite large investments [10]. Secondly, the lack of adequate digital literacy among those groups that would benefit most from public health programs, such as elderly people. The resulting digital divide would therefore be an additional cause of inequality and inefficacy [1]. Furthermore, serious concerns about patient privacy and fit with international regulations have not yet been adequately thought out. The Cambridge Analytica Data scandal in 2018 is still recent news and, despite measures such as the European Union’s General Data Protection Regulation, there is still a long way to go [11]. Lastly, the amount of information currently available on the Internet about dental treatments can lead to conflicts and misunderstandings between patients and dentists. Indeed, the management of patients’ expectations is increasingly becoming a challenge for clinicians, due to the unrealistic expectations partly due to the rise of social media [12]. There are no easy answers to these issues.

The transformation of DPH, driven by multiple factors from outside the dental profession, is inevitable. The expansion in the range of dental practice and the progressive integration oral and general healthcare will require a profound rethinking of the role of dentists in future society. The inevitability of technological advance does not deny that the community of dentists and dental specialists can have a proactive influence on this process. Indeed, when faced with future challenges, our North Star must always be the promotion of oral health in the community and among individual patients. The future depends on what we do in the present.

The aim of this Special Issue is to attempt to shed light on current challenges and issues in DPH. We hope that the readers of *International Journal of Environmental Research and Public Health* will find the articles in this Special Issue not only informative, but inspiring as well.
